# Childhood Emotional Maltreatment Severity Is Associated with Dorsal Medial Prefrontal Cortex Responsivity to Social Exclusion in Young Adults

**DOI:** 10.1371/journal.pone.0085107

**Published:** 2014-01-08

**Authors:** Anne-Laura van Harmelen, Kirsten Hauber, Bregtje Gunther Moor, Philip Spinhoven, Albert E. Boon, Eveline A. Crone, Bernet M. Elzinga

**Affiliations:** 1 Leiden University, Leiden Institute for Brain & Cognition, Leiden, The Netherlands; 2 Leiden University, Institute of Psychology, Clinical Psychology, Leiden, the Netherlands; 3 University of Cambridge, Department of Developmental Psychiatry, Cambridge, United Kingdom; 4 De Jutters, Youth Mental Health Care Center, The Hague, The Netherlands; 5 Leiden University, Institute of Psychology, Developmental Psychology, Leiden, The Netherlands; 6 Leiden University Medical Center, Department of Psychiatry, Leiden, The Netherlands; 7 De Fjord Lucertis, Centre for Orthopsychiatry and Forensic Youth Psychiatry, Capelle aan den IJssel, The Netherlands; 8 Curium-Leiden University Medical Centre, Department of Child and Adolescent Psychiatry, Leiden, The Netherlands; Tokyo Metropolitan Institute of Medical Science, Japan

## Abstract

Children who have experienced chronic parental rejection and exclusion during childhood, as is the case in childhood emotional maltreatment, may become especially sensitive to social exclusion. This study investigated the neural and emotional responses to social exclusion (with the Cyberball task) in young adults reporting childhood emotional maltreatment. Using functional magnetic resonance imaging, we investigated brain responses and self-reported distress to social exclusion in 46 young adult patients and healthy controls (mean age = 19.2±2.16) reporting low to extreme childhood emotional maltreatment. Consistent with prior studies, social exclusion was associated with activity in the ventral medial prefrontal cortex and posterior cingulate cortex. In addition, severity of childhood emotional maltreatment was positively associated with increased dorsal medial prefrontal cortex responsivity to social exclusion. The dorsal medial prefrontal cortex plays a crucial role in self-and other-referential processing, suggesting that the more individuals have been rejected and maltreated in childhood, the more self- and other- processing is elicited by social exclusion in adulthood. Negative self-referential thinking, in itself, enhances cognitive vulnerability for the development of psychiatric disorders. Therefore, our findings may underlie the emotional and behavioural difficulties that have been reported in adults reporting childhood emotional maltreatment.

## Introduction

Chronic parental rejection can be considered a core aspect of Childhood Emotional Maltreatment (CEM; emotional abuse and/or emotional neglect) [1]. For instance, during episodes of CEM, children may be ignored, isolated, or siblings may be favored. CEM has severe and persistent adverse effects on behavior and emotion in adulthood [2], and CEM is a potent predictor of depressive and anxiety disorders in later life [3], [4]. Social rejection, ranging from active isolation to ignoring basic emotional needs, may enhance sensitivity towards future rejection [5]. Along these lines, individuals reporting CEM may be especially sensitive to (perceived) social rejection. Individuals high in rejection sensitivity have a tendency to expect, perceive, and overreact to social rejection, and show enhanced distress and related neural responses to social rejection in the lab [5]. Furthermore, rejection sensitivity (both behaviourally and in terms of brain responses) is positively related to the development and maintenance of depression, social anxiety, and borderline personality disorder symptoms [6], [7]. Therefore, enhanced distress and neural responses to (perceived) social rejection may be one of the mechanisms through which a history of CEM may predispose individuals to the development of depressive and anxiety disorders in later life. However, the subjective and neural responses to social rejection in individuals reporting CEM are currently unknown.

Social rejection in the lab has been examined most frequently with the Cyberball task [8], [9]. During an fMRI compatible variation of the Cyberball task, participants play two games of virtual toss with two other players (computer controlled confederates). In the first (inclusion) game, participants are thrown the ball an equal number of throws as compared to the other players. However, in the second (rejection/exclusion) game they may receive the ball once or twice in the beginning of the game, but thereafter never receive it again. Social exclusion during the Cyberball task induces a cascade of negative emotions, including anxiety, depression, reduced sense of belonging and meaningful existence, and a reduced sense of control, and lowered self-esteem [5], [10]–[13].

Neuroimaging studies have revealed a set of brain regions that are typically activated during social exclusion in the Cyberball task, primarily in cortical midline structures; the anterior cingulate cortex (ACC)/medial prefrontal cortex (mPFC), and Insula [14], [15]. The ACC and mPFC are vital for expectancy-violation, error-detection, the processing of cognitive conflict, and self- and other referential processing [16]–[18]. In line, a recent meta-analysis suggested that activation in these regions during social exclusion might be related with enhanced social uncertainty, social distress, and social rumination [15]. Activation in the dorsal ACC/mPFC and Insula have been related to self-reported distress during exclusion in the Cyberball game, however, not all studies found dorsal ACC/mPFC responsivity to social exclusion [14], [15], [19], [20], or only found it in the first trials of the exclusion game [11]. Furthermore, studies investigating adolescents and children found *ventral* ACC/mPFC responses to distress during social exclusion [11], [21]–[23]. Increased *dorsal* ACC/mPFC to exclusion may be dependent on individual differences. As dorsal mPFC activity is especially pronounced in individuals sensitive to interpersonal rejection [24], [25], anxiously attached [26], and/or having low self-esteem [27], [28]. Therefore, dorsal ACC/mPFC responsivity to social rejection may also be evident in individuals with CEM. However, CEM related brain functioning during social exclusion has not yet been examined.

We examined the impact of a history of CEM on brain functioning and emotional distress to social exclusion. We compared young adult patients reporting a moderate to extreme history of CEM (*N* = 26) with healthy controls (*N* = 20) reporting low to moderate CEM. We examined whole brain responses while specifying the mPFC, ACC and Insula as regions of interest (ROIs) because of their important role in social exclusion [14], [15]. We hypothesized that individuals reporting a history of CEM would show enhanced brain responses and emotional distress to social exclusion. Therefore, we hypothesized that the severity of CEM would show a dose-response relationship with self-reported distress and brain responsivity.

## Methods

### Ethics statement

All participants 18 years of age or older provided written informed consent. For participants that were under 18 years of age at the time of scanning, parental/legal guardian written consent was obtained. This study was conducted according to the principles expressed in the Declaration of Helsinki, and was approved by the Leiden University Medical Center Medical Ethics committee. All participants had uncompromised capacity to consent (i.e. exclusion criteria for our study included difficulty understanding the Dutch language, or a IQ < 80).

### Sample

We included a total of 26 out- and inpatients reporting moderate to extreme CEM (‘CEM group’) who were in treatment at a center for youth specialized mental health care in the Hague, the Netherlands (mean age = 18.31 years, SD = 1.23; 6 males) and 20 healthy controls reporting low to moderate CEM (mean age = 18.85, SD = 1.95; 6 males). The CEM and control groups were matched in terms of age (*F(*1,44) = 1.38, *P = *.25), gender (*X* ^2^(1) = .28, *P = *.74), and IQ (*F(*1,44) = 2.76, *P = *.10) (see Table 1). In the CEM group, 11 patients reported regular use of anti-depressant and anti-anxiogenic medication (n = 8 used SSRI’s, n = 1 used the tricyclic antidepressant (TCA)  =  amitrypteline, and n = 3 used benzodiazepam).

**Table 1:** Demographics for the Control (n = 20) and CEM (n = 26) groups.

	Controls (n = 20)		CEM (n = 26)				
	Mean	SD	Mean	SD	Chi-Square	F	*P*
Gender M/F	6/14		6/20		.281		0.74
IQ	111.5	9.54	107.0	8.76		2.76	0.10
Age	18.85	1.90	18.31	1.23		1.38	0.25
Emotional Abuse	5.2	0.89	11.81	4.20		47.70	0.00
Emotional Neglect	6.85	1.76	17.65	3.60		151.81	0.00
Physical Abuse	5.00	0.00	6.38	2.65		5.41	0.03
Physical Neglect	4.05	0.22	6.77	3.90		9.64	0.00
Sexual Abuse	5.45	1.00	9.15	2.66		34.75	0.00

Patients in the CEM group were excluded when they had a comorbid pervasive developmental disorder or psychosis (as measured with the SCID-I [29]). In addition, current substance abuse was also set as an exclusion criterion. Current substance abuse was measured through random urine samples that are mandatory for individuals admitted at the center.

Fifteen participants from the control group had participated earlier in a study on developmental differences in neural responses during social exclusion [11]). Twenty-six participants who were >15 years of age at the time of scanning in the Gunther Moor et al. study, and who had indicated that they could be approached for future research were contacted. Twenty-one participants agreed to participate and completed the Childhood Trauma Questionnaire (CTQ [30]). Five participants were excluded based on CTQ scores indicating a history of childhood abuse; two reported moderate to severe physical abuse (both scored 12), two reported severe emotional neglect (both scored 19), and one participant reported borderline moderate/severe emotional neglect (14). To further obtain a good match with the CEM group, five control participants were recruited from the general public through an recruitment website, and through adevertisements. All control participants included in this study indicated no history of psychiatric disorder, were not taking any psychotropic drugs and had scores of low-moderate emotional abuse (<12), emotional neglect (<14), and physical neglect (<10), and no physical abuse (<6), and sexual abuse (<6), on the CTQ, according to the cut offs [30] for low severity of abuse: emotional abuse: ≥ 9; emotional neglect: ≥10; physical neglect: ≥ 8; physical abuse: ≥ 8; and sexual abuse: ≥ 6.

Finally, exclusion criteria for all participants were left-handedness, or general contra-indications for MRI, such as metal implants, heart arrhythmia, and claustrophobia, difficulty understanding the Dutch language, or a IQ< 80 (all participants completed the WAIS, or if <18 years the WISC intelligence subscales similarities and block design [31], [32]).

### Assesment of Psychopathology

In all patients with a history of CEM, DSM-IV axis I (psychiatric disorders) and DSM-IV axis II disorders (personality disorders) were assesed using the Structured Clinical Interview for DSM Disorders (SCID-I & SCID-II [29], [33]; please note that two patients in the CEM group had no SCID-I data). All patients in the CEM group had at least one axis I disorder (18 participants had multiple axis I disorders), and 19 participants had a concurrent axis II personality disorder (see Table 2 for all axis I and II diagnoses). Control participants over the age of 18 at the time of scanning reported no history of neurological or psychiatric disorders.

**Table 2:** Clinical characteristics of the CEM group.

SCID I	Depression	Alcohol abuse	Social phobia	Obsession	Generalized Anxiety	PTSD	
# current	16		10	2	1	10	
# Lifetime	9	3	4	1		3	
Total	24	3	14	3	1	13	

SCID II*	Avoidant	Dependent	Obsessive	Depressive	Passive Aggressive	Paranoid	Borderline
	11	2	3	10	1	5	7

*Note*. SCID II data for 2 participants was missing.

Control participants who were under the age of 18 at the time of scanning were screened for psychiatric disorders using the Child Behavioural Checklist (CBCL [34]) that was filled in by their parents. Control participants were only included in this study if they scored in the normal range of the CBCL (see Achenbach; 34). Control participants over the age of 18 at the time of scanning were screened for DSM-IV axis II personality disorders with the Dutch Questionnaire for Personality Characteristics (VKP [35]; Vragenlijst voor Kenmerken van de Persoonlijkheid). Because the VKP is know to be overly inclusive [35], controls with a score that indicated a ‘probable’ personality disorder on the VKP (n = 8) were also assessed with a SCID-II interview by a trained clinical psychologist (K.H.). All controls that were followed up with the SCID-II were free from personality disorder diagnoses.

### Childhood Emotional Maltreatment

History of childhood emotional maltreatment was assessed using the Dutch version of the Childhood Trauma Questionnaire (CTQ [30], [36]). In the Dutch version of CTQ, a total of 24 items are scored on a 5-point scale, ranging from *1 = never true* to *5 = very often true.* The CTQ retrospectively assessed five subtypes of childhood abuse: emotional abuse, sexual abuse, physical abuse, emotional neglect and physical neglect. The CTQ is a sensitive and reliable screening questionnaire with Cronbach’s alpha for the CTQ subscales varying between. 63-.91 [37].

In line with the American Professional Society on the Abuse of Children [1] and our previous studies on CEM [38], [39], emotional maltreatment in childhood was defined as a history of emotional neglect and/or emotional abuse before the age of 16 years. In line with the American Professional Society on the Abuse of Children [1] definition of emotional abuse that specifies that emotional abuse consists of parental isolating, intimidating, terrorizing, blaming, belittling, degrading, denying emotional responsibility or otherwise behaviour that is insensitive to the child’s developmental needs, or can potentially damage the child emotionally, or psychologically and our previous studies on CEM [38], [39], CEM was defined as a history of emotional neglect and/or emotional abuse before the age of 16 years. In line with the idea that emotional abuse rarely occurs alone [40], in our sample, there was a significant correlation between emotional abuse and emotional neglect scores (*r* = .54, p<.001), and only three participants reported emotional abuse in isolation (i.e. they reported emotional abuse that was in the moderate to extreme range (CTQ scores>12) together with only emotional neglect that was in the moderate range (CTQ scores of 11,12, and 13 on emotional neglect). As only 3 individuals reported emotional abuse in isolation we were unable to perform separate analyses for the different emotional maltreatment types.

**Figure 1:**
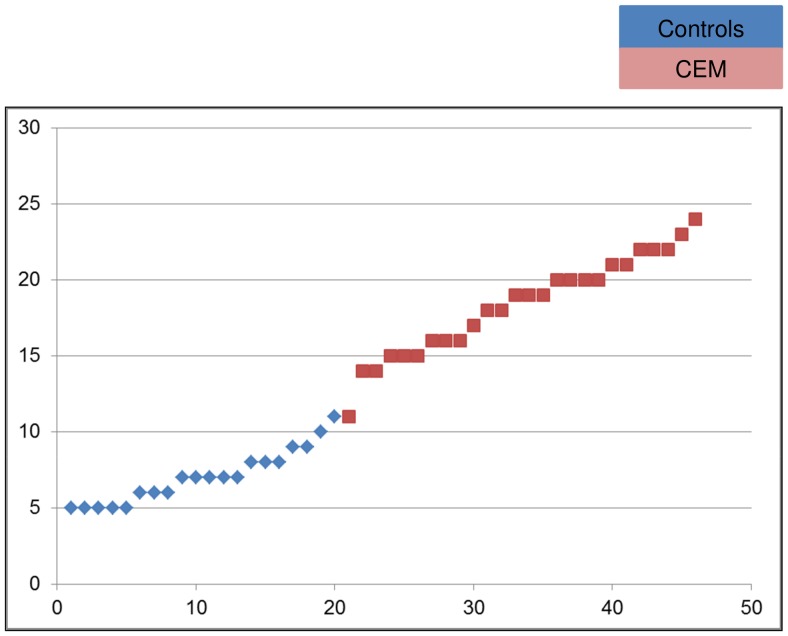
Distribution CEM severity across participants.

For the entire sample, overall CEM score was defined as the highest score on the emotional abuse or emotional neglect subscale of the CTQ (e.g., if emotional abuse score was 19, and emotional neglect score was 14, overall CEM score was 19). In our study, Cronbach’s alpha for the emotional abuse subscale was.88, for the emotional neglect subscale.94, and for the combined emotional abuse and neglect subscales.83. The CEM group reported significantly higher levels of childhood abuse compared to controls on all subscales of the CTQ (all F’s>5.41, P’s<.03), see Table 1. Self-reported CEM ranged from low to extreme CEM across participants (see Figure 1). In the control group self-reported severity of CEM ranged from low to moderate, whereas in the CEM group severity of CEM ranged from moderate to extreme [30].

### The Cyberball game

In the Cyberball game [8], [9] participants played a game of virtual toss with two other players (computer controlled confederates), depicted using animated avatars. Participants were led to believe that the other players (one female, one male) played the game online on the internet. Fictitious names of the players (common Dutch names, counterbalanced between participants) were displayed on the screen just above their avatars (i.e. in the left and right hand corners of the screen). The participant’s self was displayed on the screen as an animated hand, with the participant’s name displayed just below the hand. In the Cyberball game, participants first played the inclusion game, followed by the exclusion game. During inclusion, participants threw the ball one-third of the total amount of throws (thus, achieving an equal number of throws as compared to the other players). During social exclusion, they received the ball once in the beginning of the game, but thereafter never received it again. Immediately after inclusion, and after exclusion, participants filled in two questionnaires that assessed their distress during the game (see below for specifics on the questionnaires). All instructions, and questionnaires were presented on the screen, and all instructions were read out loud (through the intercom) by the experimenter. Finally, and before starting the Cyberball game, participants were questioned whether they understood the instructions of the game.

Both Cyberball games consisted of a total of 30 ball tosses, and each game was administered in a separate run that lasted circa 5 minutes. The duration of each ball toss was fixed to 2 seconds. We added a random jitter interval (100–4000 ms.) in order to account for the reaction time of a real player. To further increase credibility of the Cyberball game, both games started with a loading screen that notified that ‘*the computer is trying to connect with the other players’*.

### Distress: need satisfaction and mood ratings

**Figure 2:**
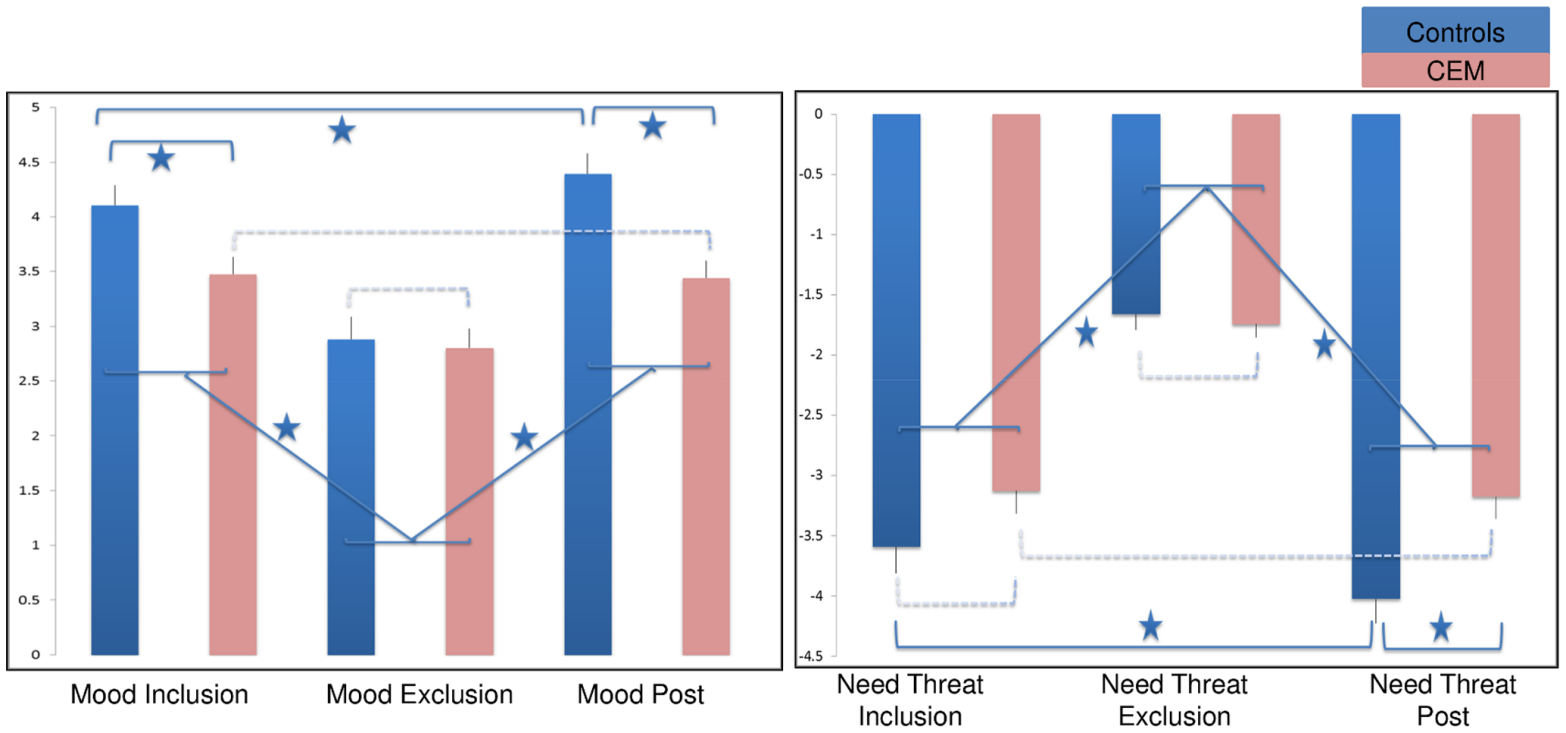
Self-reported Mood and Need threat for the Control and CEM groups. *Note*. Significant differences are indicated with an asterisk, whereas dotted lines depict non-significant differences. A high score on the Mood scale indicates high mood, whereas a high score on the Need Threat Scale indicates high need threat.

To assess distress after inclusion, exclusion, and after scanning (just before the debriefing; ‘post scanning’), all participants completed the Need Threat Scale [41], and a mood questionnaire [42]. The Need Threat Scale consists of eight items that measure self-esteem, belonging, meaningful existence, and control (each was measured with two questions). A high score on this scale indicates that the basic needs are threatened (i.e., low self-esteem, low sense of belonging to others, low sense of meaningful existence, and low sense of control). The mood questionnaire consisted of eight items that (two of each) measured feeling good/bad, relaxed/tense, happy/sad, and friendly/unfriendly. All items on the questionnaires were rated from 1 (‘not at all’) to 5 (‘very much’), and a high score on this questionnaires indicates good mood). To enhance the readability of this paper, we inverted the need threat scores (in the original scale a high need threat score indicated low need threat), which explains the negative need threat scores in Figures 2 and S2.

After inclusion and exclusion, participants were instructed to describe their mood and need threat feelings during the inclusion and exclusion game. At post-scanning, participants were instructed to assess their *current* mood and need threat feelings.

### Fmri data acquisition

Upon arrival to the lab, we first familiarized the participants with the scanning environment and sounds, using a mock scanner, and recorded scanner sounds. Actual scanning was performed on a 3.0 Tesla Philips fMRI scanner in the Leiden University Medical Center. To restrict head motion, we inserted foam cushions between the coil and the head. Functional data were acquired using T2*-weighted Echo-Planar Images (EPI) (TR  =  2.2 s, TE  =  30 ms, slice-matrix  =  80×80, slice- thickness  =  2.75 mm, slice gap  =  0.28 mm, field of view =  220). The two first volumes were discarded to allow for equilibration of T1 saturation effects. After the functional run, high-resolution T2-weighted images and high- resolution T1-weighted anatomical images were obtained.

### Fmri data analysis

Data were analyzed using Statistical Parametric Mapping (SPM8; Wellcome Department of Cognitive Neurology, London), version 8, and MATLAB 12.b. Images were corrected for differences in timing of slice acquisition, followed by rigid body motion correction. Preprocessing further included normalization to reorientation of the functional images to the anterior commissure and spatial smoothing with an 8-mm full-width half- maximum Gaussian kernel. The normalization algorithm used a 12- parameter affine transformation together with a nonlinear transformation involving cosine basic functions, and resampled the volumes to 3 mm cubic voxels. Movement parameters never exceeded 1 voxel (<3 mm) in any direction for any subject or scan. Preprocessing of the fMRI time series data used a series of events convolved with a canonical hemodynamic response function (HRF) model. In line with Gunther Moor et al. [11] BOLD responses were distinguished for events on which participants received (inclusion), or did not receive the ball (exclusion). We divided the inclusion game in three conditions; ‘receiving (‘Ball inclusion game’), not receiving and playing the ball’. During the exclusion game, the first two trials where participants received and played the ball once were not analyzed, and all other throws were set as ‘not receiving the ball (‘No-ball exclusion game’)’.

First level models were assessed using general linear model, with modeled events, and a basic set of cosine functions (to high pass filter the data) as covariates. The least-squares parameter estimates of height of the best-fitting canonical HRF for each condition were used in pair-wise contrasts. For all participants, contrasts between conditions were computed by performing one-tailed t-tests, treating participants as a random effect. To examine the effect of social exclusion and inclusion, for all analyses, we compared brain responses using the t contrast: ‘*No-ball exclusion game-Ball inclusion game*’. This contrast has previously been used Gunter Moor et al [11], where it was associated with activations in regions commonly associated with Cyberball (i.e. Insula, the ACC, and mPFC). This analysis was also performed as a t-sample t-test to examine differences between the CEM group and the control group.

Next, individual differences were added as predictors in regression analyses. First, we examined whether activation in the contrast ‘*No-ball exclusion game-Ball inclusion game*’ was associated with the self-report measurements, using whole brain regression analyses with mood, or need threat scores^II^ after exclusion (i.e. a higher score indicates a better mood, or high needs threat) as regressors of interest.

In order to examine whether the severity of CEM (see Figure 1) was related to activation in the contrast ‘*No-ball exclusion game-Ball inclusion game*’, we performed whole brain multiple regression analyses with CEM score as regressor of interest, and physical abuse, physical neglect, and sexual abuse scores as regressors of no interest. We were unable to add diagnosis (yes/no) as regressor of interest in this model, as we only had SCID II data for n = 7 controls, and no SCID II data was available for all controls. When we calculated a binary presence vs. absence variable while setting all controls at 0, there was a very high correlation between CEM score and this binary variable (r = .90). Therefore, we choose to examine the impact of Axis I and Axis II diagnosis separately within the CEM group (see Text S1), while focussing on those disorders that are known to impact responses to social exclusion (Current Depression, and Borderline Personality Disorder). Activations related to other types of maltreatment (e.g. sexual/physical abuse) during exclusion were examined with a similar whole brain multiple regression analysis, while specifying a specific type of abuse as regressor of interest, and CEM and the other types of abuse as regressors of no interest. There was multicollinearity between CEM, physical neglect, physical abuse and sexual abuse (r’s>.31, *P*,.04), however, when we repeated the regression analyses while only specifying CEM as predictor the main effects of CEM on brain activations remained unchanged.

For these analyses, brain activations were first examined at whole brain level with a threshold of *P<*.005 uncorrected, with a spatial extent K>25 voxels because this threshold and cluster extent have been suggested to provide a good balance between type 1 and type 2 errors [43]. Because of their presumed role during social exclusion, we then set the entire ACC, mPFC and Insula as Regions of interest (ROIs) (see also [14], [44]). If peak voxel activations fell within these predetermined ROIs, to further protect against Type 1 errors, we also report whether these activations were significant after small volume correction (SVC) for the spatial extent of the activated region (family wise error at the cluster level). For this SVC we used the automatic anatomical labeling (AAL) toolbox within the Wakeforest-pickatlas toolbox [45]. Brain activations where peak voxel activations fell outside our predetermined ROIs were examined at *P<*.05 FWE corrected at the whole brain level. All brain coordinates are reported in MNI atlas space. For illustration purposes, we extracted cluster activations (for the main effect of task) using the Marsbar region of interest toolbox [46].

### Behavioral analyses

Behavioral responses for the mood and need threat scales were analyzed using Group (CEM, Controls) by measurement moment (Inclusion, Exclusion, Post Scanning) Repeated Measures Analyses of Variances (ANOVAs) in IBM SPSS statistics 19. In addition, the relationship between severity of CEM across participants, and distress (mood and need threat scores) after inclusion, exclusion, and post scanning was assessed using correlational analyses. All analyses were Bonferroni corrected for multiple testing, and significance was set at *P<*.05 two-sided.

## Results

### Impact of social exclusion on self-reported mood and need threat

A Group (CEM, Controls) by measurement moment (Inclusion, Exclusion, Post Scanning) rmANOVA on mood revealed a main effect of measurement moment on mood score (*F(*2,86) = 67.47, *P<*.001), and post-hoc t-tests showed that for both groups mood scores significantly decreased from inclusion to exclusion (t’s> 5.58, *Ps<*.001), and significantly increased from exclusion to post scanning (*t*’s<–4.53, *P’s<*.001). In addition, there was a main effect of group (*F(*1,43) = 6.19, *P = *.02), and there was a significant mood × group interaction (*F(*2,86) = 9.52, *P<*.001). Figure 2 shows that after inclusion, the CEM group reported significantly lower mood scores when compared to controls (*F(*1,43) = 6.83, *P = *.012), however after exclusion, this difference disappeared (*F(*1,43) = .09, *P = *.77). At post scanning, the CEM group again reported lower mood feelings compared to controls (*F(*1,43) = 15.54, *P = *<.001).

A Group (CEM, Controls) by measurement moment (Inclusion, Exclusion, Post Scanning) rmANOVA on need threat revealed a main effect of measurement moment on need threat scores (*F(*2,88) = 162.80, *P<*.001), and post-hoc t-tests indicated that need threat scores significantly increased from inclusion to exclusion in both groups (*t*’s>9.08, *P’s<*.001), and significantly decreased from exclusion to post scanning (*t*’s>–7.80, *P’s<*.001), suggesting that exclusion in the Cyberball task significantly increased threat related feelings across participants. There was a marginal main effect of group (*F(*1,44) = 3.80, *P = *.06), and a significant need threat × group interaction (*F(*2,88) = 8.33, *P<*.001). Post-hoc tests showed that after inclusion, the CEM group reported similar need threat when compared to controls (*F(*1,44) = 2.62, *P = *.11), which remained after exclusion (*F(*1,44) = .24, *P = *.62). However, at post scanning, the CEM group reported increased need threat feelings when compared to controls (*F(*1,44) = 9.72, *P = *<.005), see Figure 2.

### Relationship between severity of CEM and self-reported distress (mood and need threat)

Across participants, correlation analyses revealed that the severity of the CEM score was negatively related to mood (*r = *–.45, *P* <.001) and positively with feelings of need threat (*r = *.29, *P<*.05) after inclusion. However, after exclusion, no relationships with CEM score and mood, nor need threat were found (*r’*s<–.02, *P*’s>.29). Finally, post scanning, CEM score was again significantly negatively related to mood (*r = *–.49, *P* <.001) and positively with need threat scores (*r = *.58, *P<*.001).

### FMRI analyses; main effect of exclusion>inclusion

**Figure 3:**
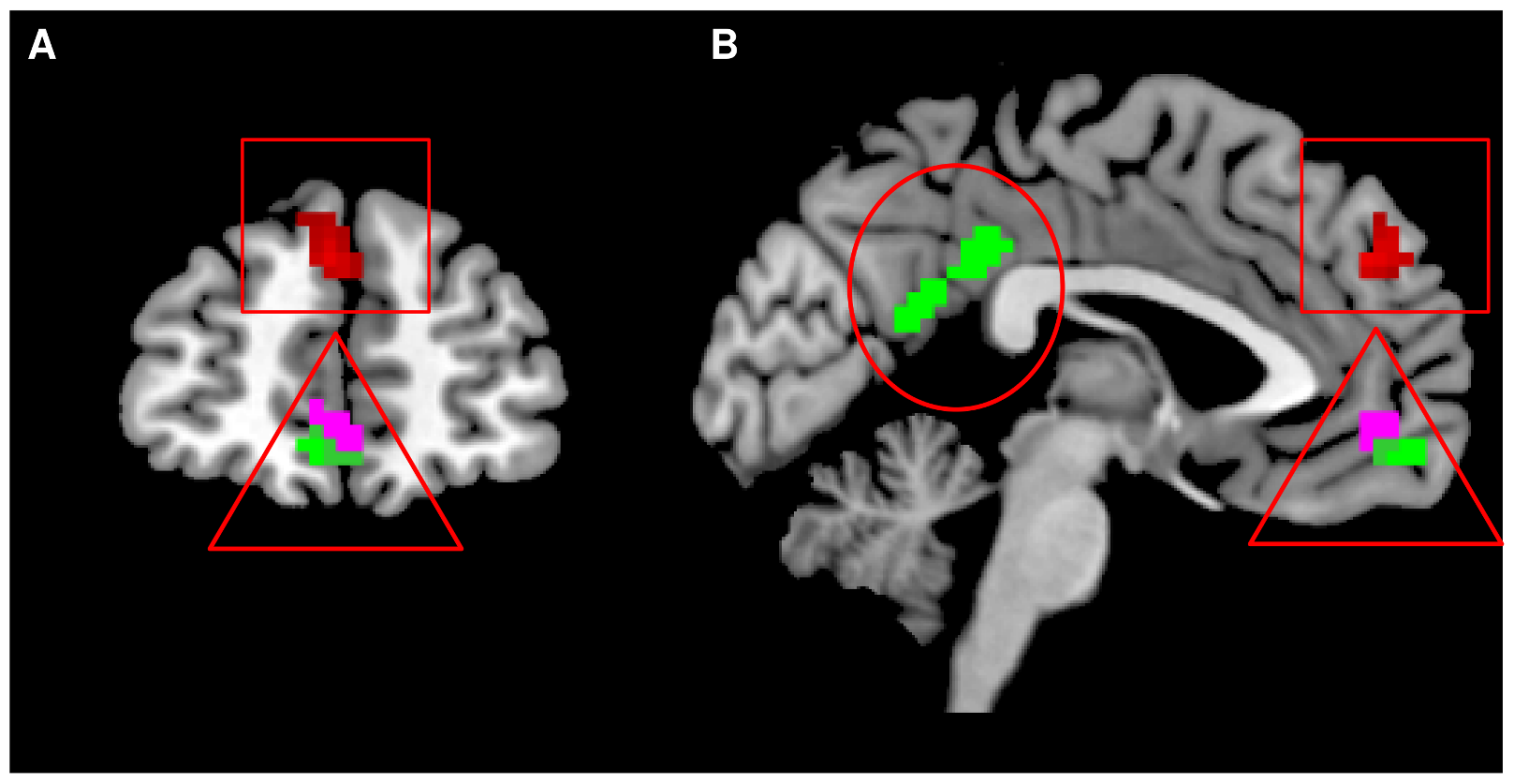
Brain responses to social exclusion (‘*No-ball exclusion game-Ball inclusion game’*) at y = –**51 (A), x = 3 (B).** *Note*. The green blobs depict the posterior cingulate (circle), and ventral mPFC cluster (triangle) that were related to social exclusion (‘*No-ball exclusion game-Ball inclusion game’*) across participants. The violet blob (triangle) depicts the ventral mPFC that was activated in response to need threat at exclusion across participants. The red blob depicts the dorsal mPFC cluster that was related to CEM across participants.

Across participants, the contrast ‘*No-ball exclusion game-Ball inclusion game’* resulted in activations in the posterior ACC (x = 0, y = –36, z = 36, K = 61, Z = 3.43, *P<*.001, (P_SVC_ = .09), and the ventral mPFC (x = –3, y = 57, z = –12, K = 44, Z = 3.51, *P<*.001, Figure 3). The activation in posterior ACC marginally survived SVC, but the ventral mPFC area did not survive SVC. All brain regions that were active at the reported threshold (*P<*.005, K>25) are presented in Table 3. An independent (CEM vs. Controls,) *t*-test in the same and the reversed contrast revealed no significant group differences.

**Table 3:** Activations for the '*No-ball exclusion game - Ball inclusion game*' contrast at P<.005, K>25.

				peak					ROI
			K	*P_FWE_*	T	Z	*P*	*x,y,z {mm}*	*P*_SVC_
Main effect across participants		Ventral mPFC	44	0.93	3.79	3.51	0.000	–3 57 –12	1.00
				1.00	3.15	2.98	0.001	6 57 –9	
				1.00	2.97	2.82	0.002	–9 45 –9	
		Posterior ACC	61	0.97	3.69	3.43	0.000	0 –36 36	0.09
				0.99	3.52	3.29	0.000	–6 –54 18	
		Inferior frontal gyrus	36	0.98	3.61	3.37	0.000	–42 27 15	
				1.00	3.31	3.11	0.001	–57 24 15	
				1.00	2.98	2.83	0.002	–54 27 6	
Mood exclusion	positive relationship	No significant clusters							
	negative relationship	Frontal inferior Opperculum	35	1.00	3.31	3.11	0.001	54 9 27	
Need treat exclusion	positive relationship	ventral mPFC	31	0.92	3.81	3.53	0.000	–3 51 –6	ns
	negative relationship	No significant clusters							
CEM vs Controls	CEM> Controls	Superior frontal gyrus	51	0.78	4.04	3.71	0.000	–24 24 51	
				1.00	2.84	2.70	0.003	–36 15 51	
		Angular gyrus	64	0.99	3.53	3.29	0.000	–51 –69 27	
				1.00	3.09	2.93	0.002	–42 –69 36	
				1.00	2.87	2.74	0.003	–33 –78 42	
	Controls> CEM	No significant clusters							
CEM severity	Negative	Superior Frontal Gyrus	56	0.71	4.15	3.77	0.000	–18 30 51	
		Dorsal Medial PreFrontal cortex	80	0.92	3.85	3.53	0.000	–3 48 33	0.05
				0.98	3.62	3.35	0.000	–12 48 42	
				1.00	2.97	2.81	0.002	6 60 30	

### Impact of CEM severity on brain activations during exclusion across participants

**Figure 4:**
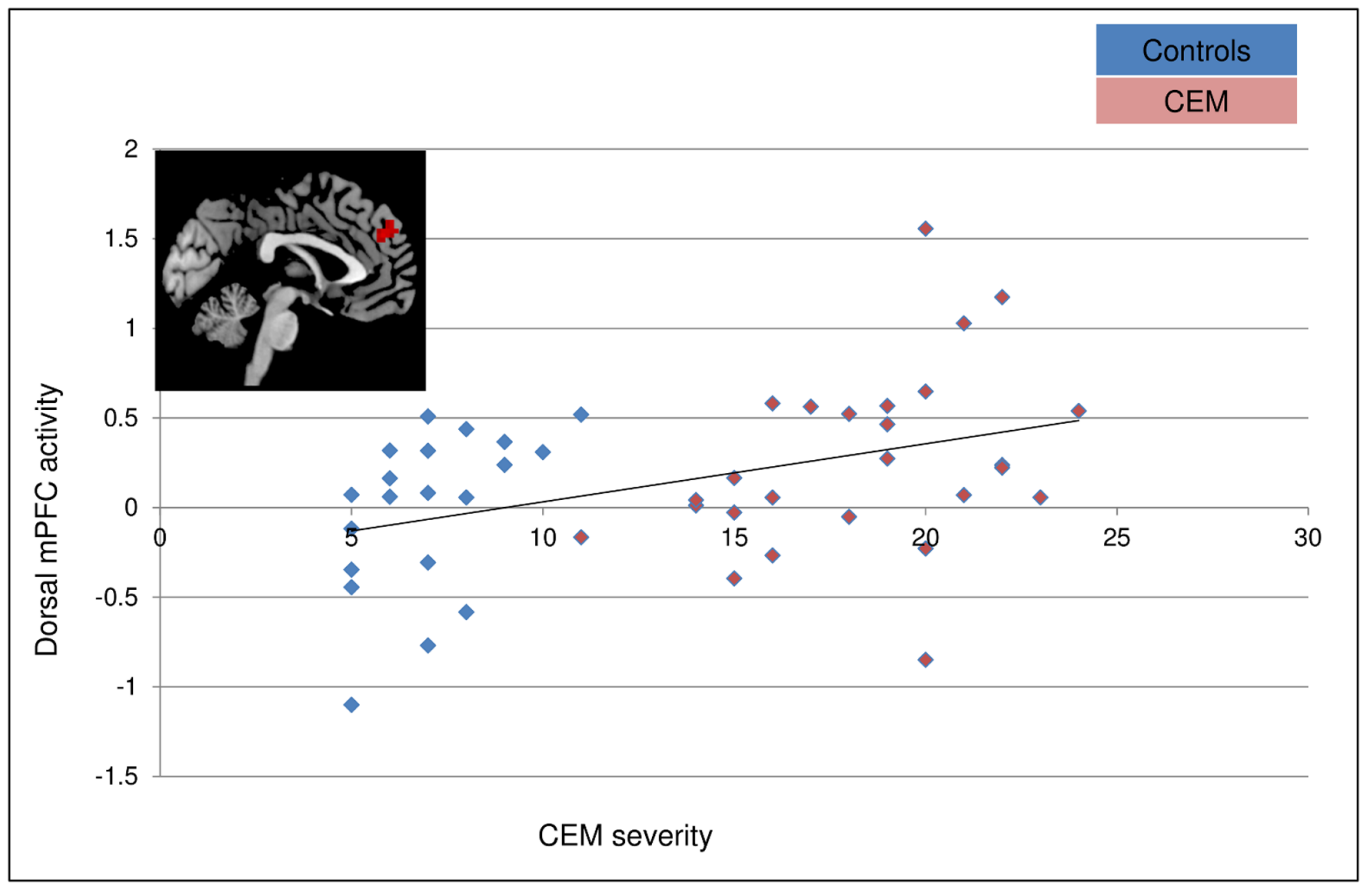
Relationship dorsal mPFC and CEM severity.

A whole brain regression analysis across all participants indicated that in the contrast ‘*No-ball exclusion game-Ball inclusion game’* the severity of CEM score had a positive association with dorsal mPFC activation (x = –3, y = 48, z = 33, K = 80, Z = 3.53, *P<*.001, (*P*_SVC_ <.05) (see Figures 3 and Figure 4). Interestingly, both within the control and CEM groups, dorsal mPFC activity in the same cluster was related with CEM severity (see Table S1, Figure S1). There were no significant negative brain activations (see Table 3), nor were there any brain activations related to physical abuse, physical neglect, nor sexual abuse for the contrast ‘*No-ball exclusion game-Ball inclusion game’*.

### Correlational analyses between distress and dorsal mPFC activation

Correlational analyses between activations in the dorsal mPFC cluster (x = –3, y = 48, z = 33), and self-reported Need Threat revealed a marginal positive relationships after inclusion (*r = *.26, *P = *.08), but not after exclusion, nor post measurement (*r’*s<.17, *P’*s>.25). Similar correlational analyses revealed that the dorsal mPFC activation was not related to self-reported mood at any of the measurement moments (*r*’s<–.23, *P’s>*.14).

### Brain activations related to distress across participants

A whole brain regression analysis indicated that need threat scores after exclusion were related to activation in the ventral mPFC contrast ‘*No-ball exclusion game-Ball inclusion game’* (x = –3, y = 51, z = –6, K = 31, *P<*.001), however, this did not survive SVC (*P*_SVC_ = 1) (Figure 3). The reversed contrast did not result in any significant differences in brain activation. Additionally, self-reported mood scores after exclusion were not associated with significant brain activations (positively, nor negatively) in the contrast ‘*No-ball exclusion game-Ball inclusion game*’.

## Discussion

We examined whether individuals reporting CEM showed enhanced neural responses and emotional distress to social exclusion. We found a dose-response relationship between the severity of CEM and dorsal mPFC responsivity to social exclusion across participants, both in individuals reporting CEM and healthy Controls. Contrary to our expectations, we did not find differences in neural responses to social exclusion when comparing patients reporting moderate to extreme CEM with Controls reporting low to moderate CEM.

Across participants, we found that social exclusion was associated with increases in posterior ACC and ventral mPFC. Although the ventral mPFC response was not significant after small volume correction, ventral mPFC/ACC responsivity to exclusion is reported by numerous studies in adolescents and children [11], [21], [23], [47]. Interestingly, the ventral mPFC and posterior ACC have been implicated in a model for self-referential processing [48]; the posterior ACC is involved in the integration of autobiographical memory with emotional information about the self [48]. Whereas, the ventral mPFC is assumed to play a role in the more affective components of self-referential processing, through emotional appraisal of self-relevant information and the coupling of emotional and cognitive processing during self-referential processing [48]. In line with the more affective role of the ventral mPFC, we found that increases in self-reported needs threat after social exclusion (i.e. reduced self-esteem, sense of belonging, meaningful existence, and control) were positively related with ventral mPFC responsivity, albeit at sub-threshold level. Taken together, our findings of posterior ACC and ventral mPFC response during social exclusion suggest that social exclusion led to negative self- and other referential processing in our sample.

Social exclusion was related to decreases in mood, and increases in needs threat in our sample, which is in line with the idea of enhanced negative self-referential processing related to social exclusion in our participants. The CEM group reported lower mood after inclusion, and at post measurement, yet after exclusion there was no significant difference between the CEM and Control group. In line, the severity of a history of CEM was negatively related with mood after inclusion; however this relationship disappeared after exclusion. These findings may be due to a floor effect in self-reported mood scores, i.e. participants could only rate their distress on a 1–5 scale, and the CEM group already reported lower mood at inclusion, leaving them little space for further reductions. The CEM group also reported higher needs threat at post-measurement, whereas the need threat scores were not significantly different from the control group during in- or exclusion, even though both groups reported an increase in need threat after exclusion. Apparently, need threat feelings were restored at post measurement in the control group, whereas in the CEM group need threat remained relatively high. These findings suggest that, at least for needs threat, the control group seems to recover quicker in the aftermath of social exclusion compared individuals with CEM. Indeed, the severity of CEM was positively related with needs threat after inclusion and at post-measurement. These findings suggest that the CEM group may show persistent negative self- and other- referential processing at post-measurement level, which was also evident after inclusion, suggesting chronic negative self-referential processing in the CEM group. This is in line with findings of our research group that CEM is associated with more negative self-cognitions [38], and more frequent self and other referential processing (i.e. more intrusions of autobiographical interpersonal memories) [39].

We found that the severity of CEM was positively related with dorsal mPFC responsivity to social exclusion. CEM related dorsal mPFC responsivity may reflect a further increase in negative self-and other-referential processing in these individuals, since the mPFC is pivotal in self-referential processing [20], [48]–[53]. And a recent meta-analysis suggested that dorsal mPFC responsivity to social exclusion is related with enhanced social uncertainty, social distress, and social rumination [15]. Dorsal mPFC in the self-referential processing model [48] has been suggested to be important for the evaluation and decision making of self-and other referential information (the evaluation whether information is relevant to the self). Therefore, our findings suggest that severity of CEM may be associated with a further increase in negative self-and other referential thinking during social exclusion. Perhaps individuals reporting CEM perceive social exclusion as especially relevant to themselves. Moreover, negative self- referential processing enhances (negative) bias and recall, resulting in more frequent, and more intense negative experiences, which in its turn enhances the negative self-referential cognitions [54]. This is consistent with the slower recovery in the CEM group, and with our previous findings of more negative and more frequent self and other referential processing in CEM [38], [39].

The finding of CEM related dorsal mPFC activity is of interest since animal studies utilizing paradigms that closely resemble CEM (e.g. maternal isolation/separation or isolation rearing) show that the mPFC is particularly affected by early life emotional stress [55]–[60]. In line, patients and healthy controls reporting CEM show a reduction in dorsal mPFC volume [61]–[63], and dorsal mPFC hypo-activity during higher order cognitive processing [unpublished data]. Therefore, our findings that individuals reporting CEM show enhanced dorsal mPFC responsivity during interpersonally stressful situations, suggest altered regulation/fluctuations of dorsal mPFC activity in individuals reporting CEM. Perhaps these findings resemble attenuation (mPFC hypo-activity) or increases (mPFC hyperactivity) in negative self- and other-referential processing in these individuals. Future studies should examine this.

Dorsal mPFC responsivity to social stress has been found to be predictive of current, and future depressive symptoms in healthy young adolescents aged 12–14 years old [7]. However, in our study we did not find that the CEM related dorsal mPFC responsivity was more prominent in our patient sample, nor was it related to a diagnosis of current depression. Across participants, mPFC responsivity was not related with self-reported mood or needs threat (although mPFC responsivity was only related with needs threat in the CEM group). Thus, our findings of CEM related enhanced mPFC responsivity in individuals with CEM may not be related with current (psychiatric) distress. Rather, these findings are more in line with the idea that increased negative self-and other referential thinking (dorsal mPFC) constitutes a vulnerability or sensitivity factor, that may underlie the emotional and behavioral vulnerabilities that have been reported in these individuals [64], [65]. And, only in interaction with other risk factors such as exposure to more recent adverse events, genetic make-up, or low social support, will this vulnerability eventually *lead* to psychopathology in later life [66].

The main effects of brain activations related to social exclusion in our sample were relatively weak. This may be related to the fact that we used the contrast ‘*No-ball exclusion game-Ball inclusion game’* in order to calculate brain activations for social exclusion. The CEM group already reported lower mood at inclusion, and we found no reduction in self-reported needs threat, nor mood in the CEM group when compared to Controls after social exclusion. This suggests that social exclusion in our sample predominantly seemed to cause distress in the control group. In addition, because the CEM group already reported relatively low mood after inclusion, the social exclusion appeared to have a relatively little further impact on self-reported distress within the CEM group. In other words, even though the CEM group may be highly sensitive to social exclusion, they may also be chronically stressed. In that sense, additional social stress may therefore not further increase brain activations related to distress during social exclusion in these individuals. Therefore, including the CEM group when examining overall brain responses related to social exclusion (‘*No-ball exclusion game- Ball inclusion game’*) in our sample may have led to a reduction in those brain responses. This may also have blurred the overall brain responses to social exclusion.

Finally, contrary to our expectations, we found no group effects on brain activations to social exclusion when comparing the CEM group with healthy Controls. This may be explained by the fact that the CEM group reported moderate to extreme CEM, and the healthy Controls reported low to moderate CEM. Whereas, we found that the severity of CEM showed a positive association with dorsal mPFC responsivity. Therefore, low-moderate CEM in the control group may have reduced our chances of finding group differences, at least in dorsal mPFC responsivity. Moreover, the CEM and Control groups did not show subjective differences in self-reported distress during exclusion, which may have further reduced our chances of finding group differences in brain functioning.

There are some limitations that need to be addressed. First of all, although current Axis I depressive diagnosis, was not related to activations in the dorsal mPFC, we could not disentangle the effect of current depression from that of history of CEM in our analyses due to high multicollinearity. Although, the findings of CEM related dorsal mPFC responses to exclusion were found across participants, and were even apparent in the Control group, suggesting that an Axis I depressive diagnosis might not confound our findings. However, to better disentangle the impact of CEM from the impact of depressive diagnosis on brain functioning during social exclusion, future studies examining patients with depression with and without CEM, and controls with and without a history of CEM are needed.

Second, in our study we assessed CEM retrospectively, and we have to stress the relative subjectivity of self-reported CEM. Furthermore, self-reported CEM may be subject to biased recall, even though a review of studies in both patients and healthy controls showed that CEM is more likely to be under-reported than over-reported [67]. And it should be noted that the test-retest reliability of the CTQ subscales for emotional abuse and emotional neglect has been found satisfactory across different ranges of samples (i.e. college students, psychiatric patients, and convenience samples) [68]. Furthermore, in a large sample of patients and controls, it was found that retrospective recall of CEM was not affected by current mood state [4].

Third, although we assessed whether controls over the age of 18 had a history of psychiatric illnesses, they were not formally screened for DSM-IV axis I disorders. However, we found that DSM-IV axis I Current Depression, which is known to impact brain responses to social exclusion, was not related with activation in the CEM related mPFC cluster during social exclusion. Therefore, it is not very likely that unidentified DSM-IV axis I Current Depression in the control group may have confounded the results.

## Conclusions

Taken together, we show that severity of CEM is positively related to dorsal mPFC responsivity to social exclusion in both patients with psychiatric disorders and healthy controls. The dorsal mPFC is vital for self and other-referential processing [48], [69]. Together with findings of more negative and more frequent self-referential processing in CEM [38], [39] and slower recovery in terms of need threat after the social exclusion task, our findings suggest increased dorsal mPFC activity during social exclusion may be related to more negative self-and other-reflective thinking in individuals reporting CEM. Increased negative self-and other referential thinking (dorsal mPFC) enhances vulnerability to the development of psychiatric disorders [54]. Therefore, our findings may be important in understanding the emotional and behavioral problems that has been reported in these individuals in adulthood [64], [65].

## Supporting Information

Figure S1 **Overlap in MPFC activations for CEM severity.** *Note.* Figure S1 depicts dorsal mPFC responsivity related to CEM severity across participants (Red), controls (Blue), and patients (yellow). Blurred colours indicate overlap between the regions.
(TIF)

Figure S2 **MPFC activations for CEM (Red) and Borderline personality (Blue).**
(TIF)

Figure S3 **Relationship mPFC and Needs Threat.** *Note*. A low score on the need threat scale indicates *low* need threat.
(TIF)

Table S1 **All brain activations related to social exclusion in the post-hoc analyses.** *Note*. CEM =  Childhood Emotional Maltreatment.
(DOCX)

Text S1 (DOCX)
