# Supplementary figures and images for: Childhood Emotional Maltreatment Severity Is Associated with Dorsal Medial Prefrontal Cortex Responsivity to Social Exclusion in Young Adults
Source: PLoS One. 2014 Jan 8;9(1):e85107. doi: 10.1371/journal.pone.0085107 (PMC3885678; doi:10.1371/journal.pone.0085107)

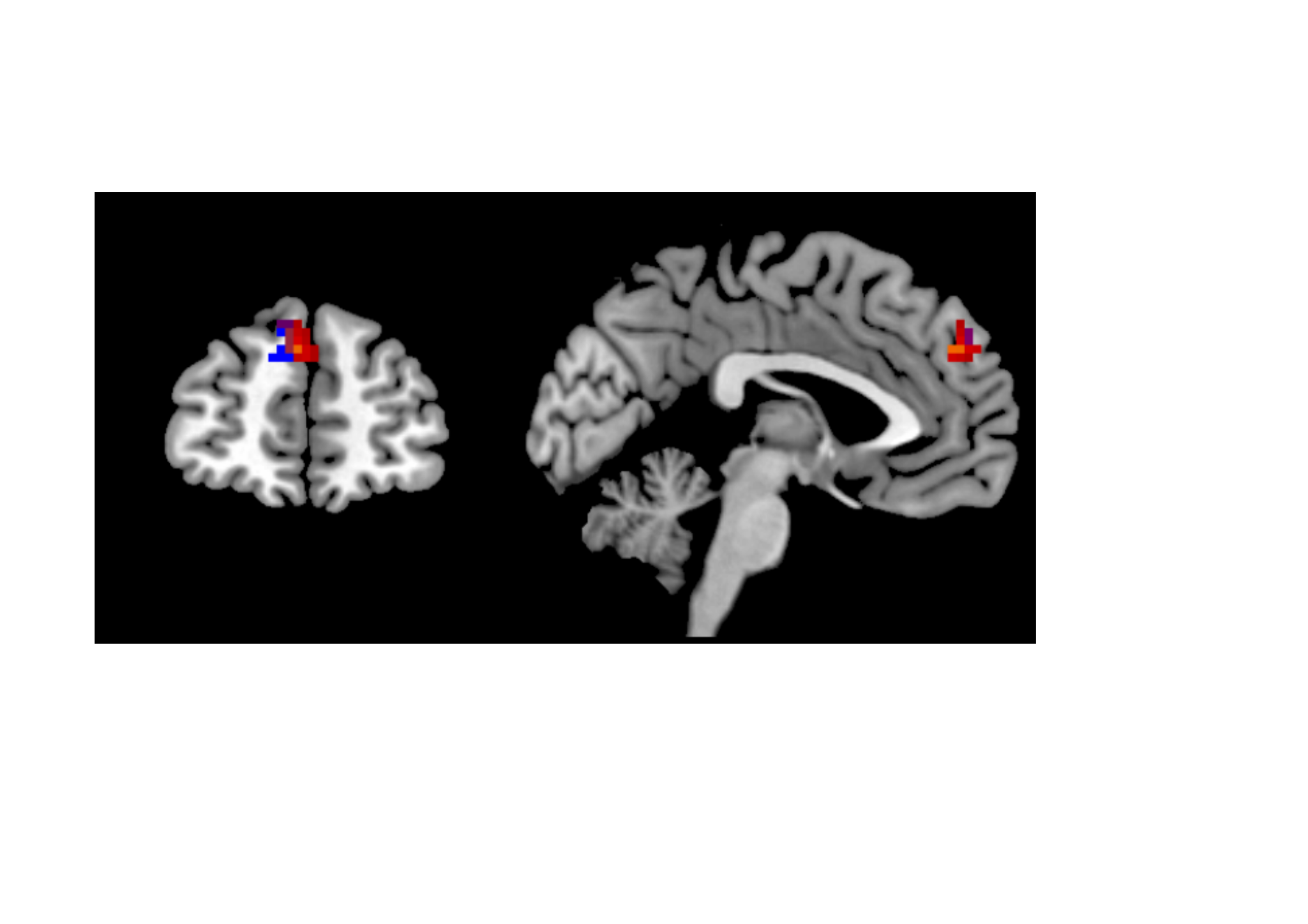

Supplement: Figure S1 — Overlap in MPFC activations for CEM severity. Note. Figure S1 depicts dorsal mPFC responsivity related to CEM severity across participants (Red), controls (Blue), and patients (yellow). Blurred colours indicate overlap between the regions. (TIF) [file pone.0085107.s001.tif]

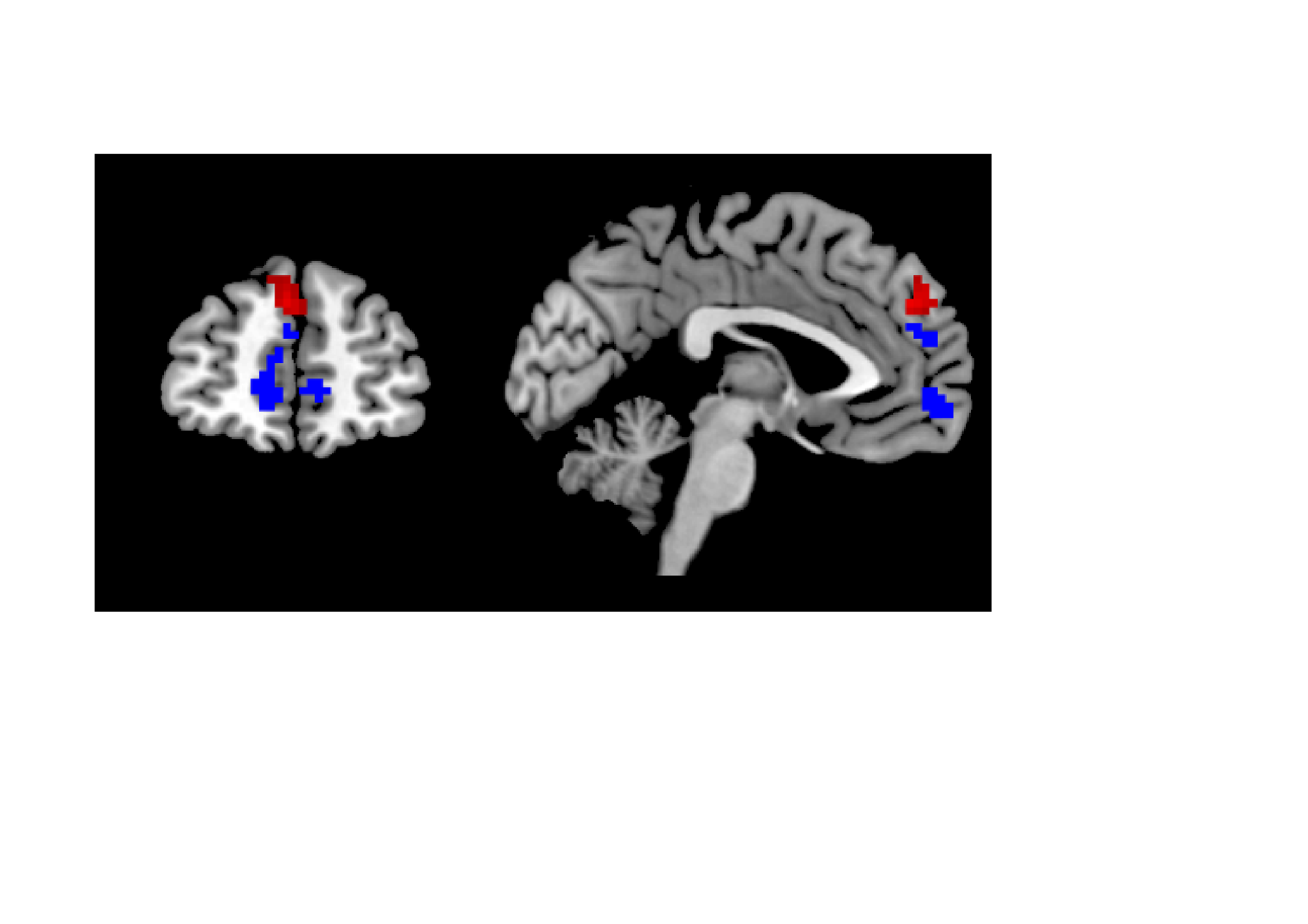

Supplement: Figure S2 — MPFC activations for CEM (Red) and Borderline personality (Blue). (TIF) [file pone.0085107.s002.tif]

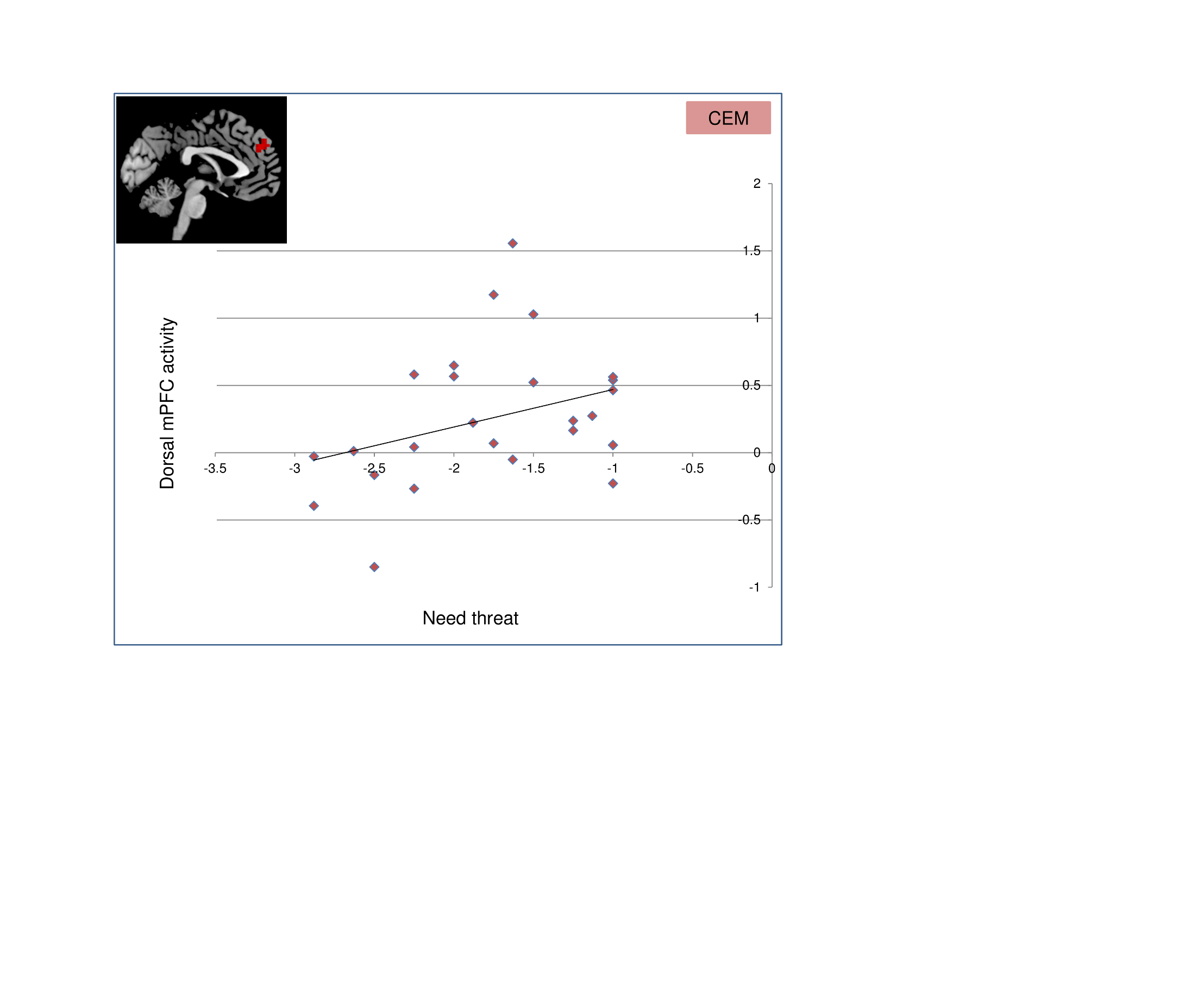

Supplement: Figure S3 — Relationship mPFC and Needs Threat. Note. A low score on the need threat scale indicates low need threat. (TIF) [file pone.0085107.s003.tif]
