# Supplementary material for: Childhood Emotional Maltreatment Severity Is Associated with Dorsal Medial Prefrontal Cortex Responsivity to Social Exclusion in Young Adults
Source: PLoS One. 2014 Jan 8;9(1):e85107. doi: 10.1371/journal.pone.0085107 (PMC3885678; doi:10.1371/journal.pone.0085107)
Supplement: Table S1 — All brain activations related to social exclusion in the post-hoc analyses. Note. CEM = Childhood Emotional Maltreatment. (DOCX) [file pone.0085107.s004.docx]

| **Table S1. All brain activations related to social exclusion in the post-hoc analyses.** | | | | | | | | |
| --- | --- | --- | --- | --- | --- | --- | --- | --- |
|  |  |  |  |  |  | peak |  |  |
| No ball exclusion game - Ball inclusion game contrast at P<.005, K>25 | | | K | P fwe | T | Z | P | x,y,z {mm} |
| Control group | CEM score | Dorsal Medial Prefrontal Cortex | 346 | 0.64 | 5.21 | 4.02 | 0.000 | 21 48 27 |
|  |  |  |  | 0.85 | 4.81 | 3.81 | 0.000 | 12 63 21 |
|  |  |  |  | 0.87 | 4.76 | 3.78 | 0.000 | 6 63 27 |
|  |  | Inferior Parietal gyrus | 43 | 0.73 | 5.05 | 3.94 | 0.000 | 39 -48 42 |
|  |  | Middle Temporal gyrus | 42 | 0.95 | 4.55 | 3.66 | 0.000 | 54 -21 -15 |
|  |  | Inferior Frontal gyrus triangularis | 27 | 0.99 | 4.19 | 3.46 | 0.000 | 36 24 24 |
|  |  |  |  | 1.00 | 3.50 | 3.01 | 0.001 | 45 21 27 |
|  |  | Middle Temporal gyrus | 37 | 1.00 | 4.14 | 3.42 | 0.000 | -66 -45 -6 |
|  |  |  |  | 1.00 | 3.49 | 3.01 | 0.001 | -60 -36 -12 |
|  |  | Dorsal Medial Prefrontal Cortex | 42 | 1.00 | 4.13 | 3.42 | 0.000 | -6 54 39 |
|  |  |  |  | 1.00 | 4.00 | 3.34 | 0.000 | -12 45 45 |
|  |  |  |  | 1.00 | 3.89 | 3.27 | 0.001 | -9 51 30 |
|  |  | Middle Temporal gyrus | 57 | 1.00 | 4.03 | 3.36 | 0.000 | 51 -45 -6 |
|  |  |  |  | 1.00 | 3.48 | 3.01 | 0.001 | 63 -39 -6 |
|  |  | Insula | 27 | 1.00 | 3.88 | 3.26 | 0.001 | 39 6 -15 |
|  |  | Caudate | 33 | 1.00 | 3.55 | 3.05 | 0.001 | -9 15 6 |
|  |  |  |  | 1.00 | 3.47 | 2.99 | 0.001 | 3 15 9 |
| CEM group | CEM score | Dorsal Medial Prefrontal Cortex | 28 | 0.52 | 4.80 | 3.98 | 0.000 | -9 54 39 |
|  | Medication use | Post Central gyrus | 30 | 0.988 | 3.85 | 3.36 | 0.000 | -21 -30 60 |
|  | Current Depression | No significant clusters |  |  |  |  |  |  |
|  | Borderline | Medial Prefrontal Cortex | 128 | 0.765 | 4.56 | 3.79 | 0.000 | -12 48 3 |
|  |  |  |  | 0.858 | 4.4 | 3.68 | 0.000 | -12 57 -3 |
|  |  |  |  | 0.998 | 3.72 | 3.24 | 0.001 | 9 51 0 |
|  |  | Caudate | 28 | 0.959 | 4.12 | 3.51 | 0.000 | -15 3 15 |
|  |  |  |  | 0.965 | 4.09 | 3.49 | 0.000 | -12 12 12 |
|  |  |  |  |  |  |  |  |  |

*Note*. CEM= Childhood Emotional Maltreatment.
