# Supplementary material for: Childhood Emotional Maltreatment Severity Is Associated with Dorsal Medial Prefrontal Cortex Responsivity to Social Exclusion in Young Adults
Source: PLoS One. 2014 Jan 8;9(1):e85107. doi: 10.1371/journal.pone.0085107 (PMC3885678; doi:10.1371/journal.pone.0085107)
Supplement: Text S1 — (DOCX) [file pone.0085107.s005.docx]

# Supplement

Post-hoc analyses within the Control and CEM groups

We also performed post-hoc analyses in order to test whether CEM related brain activations were present separately within the CEM group and control groups using a whole brain simple regression analysis with CEM score as regressor per group.

In addition, in the CEM group, 25 out of 26 patients had a current or history of Axis 1 diagnosis of depression (i.e. 16 patients had a DSM-IV axis 1 current depression diagnosis, see Table 2). Therefore, we examined the impact of presence vs. absence of current depression in a separate regression analysis. Using a similar simple regression analyses we also examined the impact of the presence vs. absence of borderline personality disorder, given the fact that rejection sensitivity has been related with borderline personality symptoms[1]. Finally, we also examined the impact of medication use on brain functioning during exclusion within the CEM group using a whole brain regression analysis.

All post-hoc regression analyses examined whole brain activations at *P<*.005, K>25. Because of their presumed role during social exclusion, we then set the entire ACC, mPFC and Insula as Regions of interest (ROIs) [see also 2,3]. Brain activations where peak voxel activations fell outside our predetermined ROIs were examined at *P<*.05, FWE corrected at the whole brain level. All brain coordinates are reported in MNI atlas space. The results of these analyses are summarized in Table S1.

CEM related brain activations to exclusion within the groups

In the control group, CEM score was positively associated in the contrast ‘*No ball exclusion game-Ball inclusion game*’ with activations in the right mPFC (x=21, y=48, z=27, K=346, Z=4.02, *P<*.001, and left dorsal mPFC (x=-6, y=54, z=39, K=34, Z=3.36, *P<*.001). Figure S1 shows that this was the same region where we found CEM related activations across participants. Finally, CEM score was also associated with Insula activation in the controls (x=39, y=6, z=-15, K=27, Z=3.26, *P<*.001). There were no other significant brain activations (see Table S1).

Within the CEM group, CEM score was positively associated in the contrast ‘*No ball exclusion game-Ball inclusion game*’ with activation in dorsal mPFC (x=-9, y=54, z=39, K=28, Z=3.98, *P<*.001). This is the same region that was also significantly related to CEM score across participants (see Figure S1). There were no other significant brain activations (Table S1).

Brain activations related to current depression, borderline personality or medication use

A whole brain regression analysis showed that the presence (n=16) vs. absence (n=10) of a *current* diagnosis of depression in the CEM group was not associated with any brain activations in the contrast ‘*No ball exclusion game-Ball inclusion game*’.

A similar whole brain regression analysis revealed that medication use (yes, no) was not associated with significant brain activations in the contrast ‘*No ball exclusion game-Ball inclusion game*’.

Moreover, A whole brain regression analysis showed that the presence (n=7) vs. absence (n=17) of Borderline personality disorder was associated with activations in the mPFC and caudate in the contrast ‘*No ball exclusion game-Ball inclusion game*’ (see Table S1). However, the very small size of the individuals with a BPD diagnosis severely hampers the interpretation of this finding. Moreover, this region did not overlap with the mPFC cluster that was related with CEM, and hence cannot explain these findings (Figure S2).

Relationship dorsal mPFC and self-reported distress within the groups

Within the Control group, there was no significant relationship between this dorsal mPFC activation and self-reported need threat after inclusion, exclusion, or post measurement (*rs<*.21, *Ps>*.38). Similarly, there was no relationship between dorsal mPFC and self-reported mood after inclusion, and exclusion (*rs<*.23, *Ps>*.34), however, there was a significant relationship between mood at post-measurement and dorsal mPFC responsivity (r=.44, *P=*.06).

Interestingly, within the CEM group, there was a marginal significant relationship between dorsal mPFC activation and need threat after exclusion (*r=*.34 *P=*.09, see Figure S2), but not after inclusion or at post measurement (*rs<*.21 *Ps>*.31). Furthermore, dorsal mPFC activity was not associated with mood after inclusion, exclusion, nor at post measurement (*rs<*-.19, *Ps>*.35).

Relationship CEM severity and self-reported distress (mood and need threat)

Within the CEM group, CEM score was (marginally) negatively related to mood at all measurement moments (r’s>-.37, *P’s<*.06), and positively related to needs threat after exclusion (*r=*.53, *P<*.005), but not after inclusion and at post measurement (r’s<.33, *Ps>*.10). No relationships with CEM score and mood or needs threat were found in the control group (all *rs<*.37, all *Ps>*.12).

References

1. Rosenbach C, Renneberg B (2011) Abgelehnt, ausgeschlossen, ignoriert: Die Wahrnehmung sozialer Zurückweisung und psychische Störungen – eine Übersicht. Verhaltenstherapie 21: 87–98. Available: http://www.karger.com/doi/10.1159/000328839.

2. Eisenberger NI (2012) The pain of social disconnection: examining the shared neural underpinnings of physical and social pain. Nat Rev Neurosci 13: 421–434. Available: http://www.ncbi.nlm.nih.gov/pubmed/22551663.

3. Meyer ML, Masten CL, Ma Y, Wang C, Shi Z, et al. (2012) Empathy for the social suffering of friends and strangers recruits distinct patterns of brain activation. Soc Cogn Affect Neurosci. Available: http://www.ncbi.nlm.nih.gov/pubmed/22355182.

Supplementary Tables and Figures

**Table S1. All brain activations related to social exclusion in the post-hoc analyses.**

*Note*. CEM= Childhood Emotional Maltreatment.

**Figure S1. Overlap in MPFC activations for CEM severity**

*Note.* Figure S1 depicts dorsal mPFC responsivity related to CEM severity

across participants (Red), controls (Blue), and patients (yellow). Blurred colours

indicate overlap between the regions.

**Figure S2. MPFC activations for CEM (Red) and Borderline personality (Blue).**

**Figure S3. Relationship mPFC and Needs Threat**

*Note*. A low score on the need threat scale indicates *low* need threat.
